# Acute Urticaria in Inpatients Undergoing Non-emergent Coronary Angiography With Corticosteroid Prophylaxis: A Retrospective Study

**DOI:** 10.3389/fmed.2021.616015

**Published:** 2021-06-10

**Authors:** Bangtao Chen, Fubing Yu, WenChieh Chen, Yong Wang, Fei Hao

**Affiliations:** ^1^Department of Dermatology, The Third Affiliated Hospital of Chongqing Medical University, Chongqing, China; ^2^Department of Dermatology and Allergy, Technical University of Munich, Munich, Germany; ^3^Department of Cardiology, The People's Hospital of China Medical University, The People's Hospital of Liaoning Province, Shenyang, China

**Keywords:** acute urticaria, iodinated contrast media, coronary angiography, premedication, risk factors

## Abstract

**Background and Aims:** Acute urticaria (AU) is the most frequently reported immediate hypersensitivity reaction in skin by administration of iodinated contrast media (ICM). We aimed to establish the pattern and identify the risk factors of AU among inpatients undergoing non-emergent coronary angiography (CAG) with prophylactic corticosteroids in China.

**Methods:** Medical records of 19,326 adult inpatients undergoing non-emergent CAG with prophylactic methylprednisolone in 2013–2019 were retrospectively investigated. AU was identified within 1 h post-ICM administration, and diffuse involvement was defined when wheals occur in two or more body parts, including the back, abdomen, chest, and extremities. Age- and sex-matched inpatients (1:4) without AU were randomly selected for assessment of risk factors.

**Results:** Approximately 0.8% of CAG inpatients had AU, including 101 diffuse and 64 limited form. The diffuse AU was more common in settings of non-diagnostic CAG, iohexol used, average ICM injection≥3 ml/min, recurrent CAG, and past history of immediate hypersensitivity to ICM. Inpatients with preexisting allergies, decreased evaluated glomerular filtration rate, and increased high sensitivity C reactive protein or neutrophil-to-lymphocyte ratio prior to CAG had a higher probability of AU (odds ratio >1, *P* < 0.05 for all variables). All AU inpatients complained of pruritus, and mild itching predominated. AU dissipated in several days under treatment of ebastine or levocetirizine 10 mg/daily, but ebastine showed superiority.

**Conclusions:** ICM-induced AU is not uncommon in non-emergent CAG inpatients with prophylactic methylprednisolone. Preexisting allergies, renal dysfunction, and mild inflammation are high-risk factors, and antihistamine monotherapy is a favorable candidate for ICM-related AU.

## Introduction

Iodinated contrast media (ICM) is indispensable and invaluable for enhancing the quality of imaging by improving the visibility of specific organs, blood vessels, or tissues. With recent advancements in medical-imaging equipment and ICM-enhanced image-guided diagnostic requirements, the application of ICM is constantly increasing. Nowadays, it is estimated that over 100 million ICM-requiring procedures are performed annually worldwide and are increasing by 20% annually in China (1, 2). The increase in elderly population and incidence of cardiovascular diseases have also led to the widespread use of ICM through the intra-arterial route in coronary angiography (CAG) with or without percutaneous coronary intervention (PCI) (3).

Adverse reactions to ICM, primarily as hypersensitivity reactions (HSRs), “allergic-like” reactions, or anaphylaxis, have been recognized as an important issue (4). ICM-related HSRs comprise a broad range of clinical features with involvement of nearly all organ systems. They can be generally classified as immediate or delayed HSRs, occurring within 1 h or 1 h−2 weeks post-ICM administration, respectively (5). Immediate HSRs to ICM usually show cutaneous symptoms with acute limited or diffuse urticaria (6). ICM-induced acute urticaria (AU) is occasionally very severe or even lethal, especially diffuse AU with concurrent dyspnea and facial angioedema (7, 8).

Accurate incidence of ICM-induced AU cannot be acquired from reported literature since large-scale studies have grouped all HSRs (urticarial or non-urticarial, mild, or severe) caused by intra-arterially or intravenously administrated ICM together (9–12). In addition, ICM-induced AU has long been regarded as non-IgE-mediated pseudoallergic reaction involving direct complement activation (13), while accumulating evidence indicated that ICM-specific IgE-mediated AU (type I allergic reaction) may have been underreported in the past due to lack of pertinent allergy testing (14, 15). Without regard to IgE levels, excessive histamine or other biogenic amines released by basophils and tryptase by mast cells are also a characteristic of immediate HSRs (including AU) to ICM, which were partially evidenced by antihistamine treatments and blood tests (9). However, another disputable aspect that prevention of immediate HSRs by premedication with corticosteroids or antihistamines remains uncertain still challenges the role of IgE-mediated allergy in AU pathogenesis.

The occurrence of ICM-related AU is unpredictable and varies with underlying diseases, the routes of ICM administration, prophylaxis strategies, etc. (7, 8). For all we know, no studies focused on AU in patients who are exposed to ICM administered intra-arterially in China and abroad. In this retrospective study, we aimed to determine the prevalence, pattern, risk factors, and the management of AU in inpatients on corticosteroid prophylaxis undergoing non-emergent CAG with or without PCI in China. To some extent, the findings of the present study will help dermatologists and cardiologists to better understand and manage AU.

## Patients and Methods

### Study Population

Inpatients aged ≥18 years who received non-emergent CAG from January 2013 to December 2019 at the Department of Cardiology in the People's Hospital of China Medical University were included in the study. The exclusion criteria were as follows: current pregnancy; severe heart failure [New York Heart Association (NYHA) IV]; pulmonary edema; known allergy to antiplatelet aggregation agents including aspirin, clopidogrel, or ticagrelor; known allergy to proton pump inhibitors; patients on hemodialysis or those with estimated glomerular filtration rate (eGFR) <15 ml/min/1.73 m^2^; refusal to accept interventional therapy; and life expectancy <1 year. To assess the relevant risk factors in the same period of time, a control group was retrospectively selected among patients without AU after 1:4 matching for age and sex. The study was approved by the Ethics Committee of the People's Hospital of China Medical University. No patient consent was needed due to a retrospective study and analysis on the medical records of the recruited patients.

### CAG Procedure

Three types of CAG were involved. The diagnostic referred to CAG without PCI, the coronary chronic total occlusion (CTO) interventional referred to CAG with PCI for treatment of CTO lesion, and the general interventional referred to CAG with PCI for treatment of partial occlusion lesion. A standard Judkins technique was used in all the studied cases. The CAG was performed via the radial or/and femoral artery approach. Bilateral CAG and PCI were performed when necessary. For CTO lesions, antegrade crossing techniques were preferably attempted, and retrograde crossing techniques were alternative if the former failed. The use of ICM (iohexol or iodixanol) was left to the operator's discretion. The prophylactic premedication to prevent HSRs included two doses of methylprednisolone 32 mg orally administered, 12 and 2 h before CAG, which is the standard of care in our institution. For prophylactic anticoagulation, a loading dose of aspirin 300 mg combined with clopidogrel 300 mg and heparin 100 U/kg were administered prior to and during the procedure, respectively. All patients were routinely administered with PPI for preventing the occurrence of stress ulcers. Withdrawal of potential nephrotoxic drugs and intravenous hydration conformed to the recommendations in the European Society of Cardiology guidelines. Dermatologists in our hospital were invited for cooperative consultation about the observed cutaneous symptoms or signs, and AU was diagnosed and recorded by dermatologists.

### Data Collection

Demographic data (age, gender, occupation, education background, ethnicity, height, and weight), preexisting conditions (allergic or non-allergic comorbidities, pre-procedural medication, past history of ICM exposure, and previous immediate HSRs to ICM during CAG), eGFR, inflammatory biomarkers including high sensitivity C reactive protein (Hs-CRP) and neutrophil-to-lymphocyte ratio (NLR), CAG procedure (type of CAG, ICM used, procedural access, ICM volume in total, and time of procedure), and AU-related characteristics (type, onset time, distribution of wheal, degree of itching, concurrent facial angioedema and dyspnea, the antihistamine regimen, and its efficacy) were all obtained through retrospective analysis of the electronic medical records. Limited AU was defined by the occurrence of wheals in only one of the five parts (upper extremities, lower extremities, chest, abdomen, or back), while diffuse AU in two or more locations.

### Statistical Analyses

Statistical analysis of the data compiled in an Excel databank was conducted using the SPSS/PC software (Version 19.0 for Windows; SPSS Inc., China). For descriptive analysis, the continuous and categorical variables were presented as mean (M) ± standard deviation (SD) and percentage (%), respectively. For the comparison of demographic information and clinical characteristics between groups, chi-square test or Student's *t*-test was used. Related covariates with significant associations with the occurrence of AU in the univariate analysis were resubjected to multivariate logistic regression analysis. For continuous variables, the receiver-operating characteristic (ROC) curve was depicted to calculate the cutoff value, sensitivity, and specificity of each predictor. A *P* < 0.05 was accepted as the cutoff for statistical significance.

## Results

### Incidence of AU

During the designated 7 years, a total of 24,058 non-emergent CAGs in 19,326 patients were conducted, while AU occurred in 0.8% (165/19,326) of the patients. Stratification of the AU according to ICM and procedure profiles was summarized in Table 1. Overall, more patients showed diffuse AU than limited AU (61.2 vs. 38.8%, *P* = 0.018), which was also observed in subgroups of general interventional CAG (*P* = 0.029), CTO interventional CAG (*P* < 0.001), application of iohexol (*P* = 0.018), average ICM injection speed more than 3 ml/min (*P* = 0.001), recurrent CAG (*P* = 0.048), and past history of immediate HSRs to ICM during CAG (*P* = 0.012). On the other hand, limited AU was more common only in patients undergoing diagnostic CAG (*P* = 0.014).

**Table 1:** AU distribution according to ICM and procedure profiles.

**Group or subgroup**	**Cases**,	**Limited AU**,	**Diffuse AU**,	***P*-value**
	***n***	***n* (%)**	***n* (%)**	
Total	165	64 (38.8)	101 (61.2)	0.018
**Type of CAG**				
Diagnostic	41	30 (73.2)	11 (26.8)	0.014
General interventional	46	14 (30.4)	32 (69.6)	0.029
CTO interventional	78	20 (25.6)	58 (74.4)	0.000
**Procedural access**				
Radial	73	32 (43.8)	41 (56.2)	0.389
Femoral	52	19 (36.5)	33 (63.5)	0.116
Radial and femoral	40	13 (32.5)	27 (67.5)	0.068
**ICM genera**				
Iohexol	101	36 (35.6)	65 (64.4)	0.018
Iodixanol	64	28 (43.1)	36 (56.3)	0.414
**ICM volume, ml**				
< 100	45	19 (42.2)	26 (57.8)	0.394
100–200	67	25 (37.3)	42 (62.7)	0.089
More than 200	53	20 (37.8)	33 (62.2)	0.144
**ICM volume/procedural time, ml/min**				
≤ 3	66	34 (51.5)	32 (48.5)	0.841
More than 3	99	30 (30.3)	69 (69.7)	0.001
Initial CAG	126	52 (41.3)	74 (58.7)	0.109
Recurrent CAG	39	12 (30.8)	27 (69.2)	0.048
Past IHR to ICM during CAG				
Yes	28	6 (21.4)	22 (78.6)	0.012
No	11	6 (54.5)	5 (45.5)	0.806

*CTO, coronary chronic total occlusion; IHR, immediate hypersensitivity reactions; ICM, iodinated contrast media; AU, acute urticaria*.

### Clinical Characteristics of ICM-Related AU

Between subgroups of limited AU and diffuse AU, there was no statistically significant difference in the onset time (*P* = 0.462). The wheals mostly occurred on the back (43.6%) in patients with limited AU and concomitantly on the chest and back in all diffuse AU patients. Itchiness was recorded in all AU patients. Mild pruritus was complained by 68.8% of the patients with limited and by 53.5% with diffuse AU, with no differences observed in the degree of pruritus between the two groups (*P* = 0.330, 0.257, and 0.433 for the mild, moderate, and severe, respectively). Only four and one diffuse AU cases concurrently experienced facial angioedema and dyspnea, respectively (Table 2).

**Table 2:** The clinical characteristics of AU.

**Variable**	**Limited AU**	**Diffuse AU**	***P*-value**
	**(*n =* 64)**	**(*n =* 101)**	
Onset time of AU, mean (SD), min	18.2 (9.5)	16.9 (11.6)	0.462
**The location that wheals occurred on**			
➀Upper extremities, *n* (%)	7 (10.9)	➂+➄:45 (44.6)	–
➁Lower extremities, *n* (%)	3 (4.9)	➂+➄+➃:27 (26.7)	–
➂Chest, *n* (%)	16 (25.0)	➂+➄+➀: 18 (17.8)	–
➃Abdomen, *n* (%)	10 (15.6)	➂+➄+➁: 11 (10.9)	–
➄Back, *n* (%)	28 (43.6)		–
Comorbid pruritus, *n* (%)	64 (100.0)	101 (100.0)	1.000
Mild itching, *n* (%)	44 (68.8)	54 (53.5)	0.330
Moderate itching, *n* (%)	18 (28.1)	41 (40.6)	0.257
Severe itching, *n* (%)	2 (3.1)	6 (5.9)	0.433
Comorbid facial angioedema, *n* (%)	0 (0.0)	4 (3.9)	0.114
Comorbid dyspnea, *n* (%)	0 (0.0)	1 (1.0)	0.427

*ICM, iodinated contrast media; AU, acute urticaria*.

### Risk Factors for ICM-Induced AU

Men accounted for 70.9% (117 cases) of the 165 AU patients with mean age ± SD 65.2 ± 10.5 years. In the control group, a total of 660 age- and sex-matched cases were randomly selected from the remaining 19,161 non-AU patients. Univariate and multivariate analyses showed that preexisting allergic rhinitis, bronchial asthma, antibiotics allergy, past immediate HSRs to ICM during previous CAG, a lower level of eGFR as well as a higher level of Hs-CRP and NLR prior to CAG, CTO interventional CAG, and the application of iohexol are risk factors for the occurrence of AU (*P* < 0.05 for all variables, Table 3). eGFR ≤ 69 ml/min/1.73 mm^2^ (sensitivity: 85.71%, specificity: 73.23%), serum Hs-CRP >8.0 mmol/L (sensitivity: 67.35%, specificity: 84.32%), and NLR>2.8 (sensitivity: 76.2%, specificity: 89.0%) were demonstrated to be the predictors of AU in further ROC curve analyses (*P* < 0.05 for all variables).

**Table 3:** Risk of AU and odds ratios of significant covariates.

	**Univariate analysis**			**Multivariate analysis**		
	**OR**	**95% CI**	***P-*value**	**OR**	**95% CI**	***P*-value**
Male	1.240	0.804–1.821	0.690	–	–	–
Age	0.920	0.690–1.259	0.297	–	–	–
BMI	1.301	0.708–1.558	0.408	–	–	–
Non-allergic comorbidities	1.258	0.908–2.018	0.227	–	–	–
LVEF ≤ 40%	1.726	1.039 −1.958	0.033	1.598	0.928–2.581	0.294
Anti-hypertension	1.608	1.104–1.981	0.024	1.602	0.902–1.887	0.369
Past history of CAG	1.332	0.792–1.592	0.529	–	–	–
Previous IHR to ICM	2.395	1.108–4.285	0.037	2.209	1.211–5.279	0.029
Allergic rhinitis	1.872	1.057–2.895	0.021	1.628	1.128–3.695	0.041
Bronchial asthma	1.487	1.106–2.807	0.018	1.502	1.205–2.217	0.036
Antibiotics allergy	2.512	1.122–4.225	0.024	2.197	1.442–3.659	0.020
Food allergy	1.524	0.997–1.567	0.624	–	–	–
CTO interventional CAG	2.251	1.038–4.064	0.037	2.490	1.212–5.307	0.019
Iohexol	2.012	1.210–3.981	0.028	1.987	1.124–4.027	0.022
Iodixanol	1.825	0.692–3.272	0.088	–	–	–
Fasting blood-glucose	1.943	1.192–4.318	0.021	1.261	0.264–2.191	0.371
eGFR	1.819	1.152–2.957	0.024	1.792	1.282–3.948	0.033
Hs-CRP	1.992	1.129–4.128	0.019	2.017	1.115–4.298	0.032
NLR	1.821	1.029–2.921	0.022	1.720	1.112–3.018	0.025

*BMI, body mass index; LEVF, left ventricular ejection fraction; CTO, coronary chronic total occlusion; CAG, coronary angiography; IHR, immediate hypersensitivity reactions; eGFR, evaluated glomerular filtration rate; Hs-CRP, high sensitivity C reactive protein; NLR, neutrophil to lymphocyte ratio; ICM, iodinated contrast media; AU, acute urticaria; OR, odds ratio; CI, confidence interval*.

### Management of ICM-Induced AU

A total of 26 patients with limited AU and 42 with diffuse AU patients were treated with levocetirizine (10 mg daily), and the remaining AU patients were treated with ebastine (10 mg daily). As Table 4 shows, ebastine exerted better therapeutic effect regarding pruritus regression in the two types of AU (both *P* < 0.001) and wheals dissipation in the diffuse form (*P* < 0.001) compared with levocetirizine.

**Table 4:** Recovery time for AU after antihistamines therapy [mean (SD), days].

**Items**	**Limited AU**			**Diffuse AU**		
	**Levocetirizine**	**Ebastine**	***P*-value**	**Levocetirizine**	**Ebastine**	***P*-value**
	**(*n =* 26)**	**(*n =* 38)**		**(*n =* 42)**	**(*n =* 59)**	
Wheals	2.4 (1.1)	2.1 (0.9)	0.236	3.6 (1.7)	2.3 (1.2)	0.000
Pruritus	2.5 (1.5)	1.3 (1.2)	0.000	3.9 (1.9)	2.5 (1.2)	0.000

*ICM, iodinated contrast media; AU, acute urticaria; levocetirizine, 10 mg daily; ebastine, 10 mg daily*.

## Discussion

The overall incidence of immediate HSRs to ICM ranges from 0.2 to 3% for mild-to-moderate cases and 0.004 to 0.04% for severe cases (16). Specifically, incidence associated with intravenously and intra-arterially administered ICM is 0.7–15 and 3.6%, respectively (17, 18). Although the overall safety profiles are well-understood, research on immediate ICM HSRs is still ongoing, and more results are greatly anticipated, especially those based on specific anaphylaxis or reaction.

This study was the first to focus on ICM-related AU in Asian inpatients undergoing non-emergent CAG with or without PCI and found that 0.8% (165/19,326) of the cases under prophylaxis with two doses of methylprednisolone (32 mg) experienced AU, which was roughly consistent with previous reports (14, 19). In addition, similar to what was observed in premedicated patients with exposure to intravenously administrated ICM or non-premedicated patients with exposure to intra-arterial ICM (2, 11, 18), a higher proportion of diffuse AU (61.2%) during CAG was found. Such results may further question the value of premedication with corticosteroids or antihistamines for prophylaxis of ICM HSRs including AU (20, 21). On one hand, premedications are not globally standardized. Different from that in our department of cardiology, high-risk patients in North America usually receive oral prednisone (50 mg) at 13, 7, and 1 h or diphenhydramine (1 mg/kg) at 1 h before ICM injection (22). On the other hand, premedication is not generally applied in Europe since breakthrough reactions of all severity grades have been observed in 1.2% up to 40% of pretreated patients, and pretreatment drugs themselves can occasionally induce adverse drug reactions including anaphylaxis (14, 19, 22, 23). Furthermore, a recent multidisciplinary consensus also opposed routine premedication, even in patients with prior HSRs to ICMs (24).

Although premedication may not always work, it seems that patients with preexisting high-risk factors usually urge clinicians to create more effective management approaches for ICM HSRs. In this regard, risk identification and related stratification in candidates for CAG are still critical for prophylaxis of ICM-associated AU or other immediate HSRs. Our results confirmed that atopy inpatients with immediate HSRs to ICM in previous CAG, preexisting allergic rhinitis, bronchial asthma, and antibiotics allergy are predisposed to the onset of AU during CAG, which were almost consistent with the findings from previous studies grouping all allergic reactions together in prophylactic and non-prophylactic patients (12, 18). Additionally, the present study found that decreased eGFR or inflammation with mildly elevated Hs-CRP and NLR were also risk factors for the occurrence of ICM-related AU, which has not been unmasked previously. It is well-proved that mild systemic inflammation positively correlates with ICM-caused acute renal insufficiency that is marked by gradually decreasing eGFR (25, 26). Dysfunction in kidney combined with longer and higher concentration of ICM exposure during CTO interventional CAG may further increase the risk of AU. Once the patients were suspected to be predisposed to ICM-related immediate HSRs before ICM administration, pretests including prick test with undiluted ICM and intradermal test with 1:10 dilution are recommended, which may potentially identify alternative ICM that could be well-tolerated (27, 28).

However, breakthrough reactions are sometimes unavoidable even after general premedication and careful screening. Thus, the more complicated problem is the application of remedy strategies, especially for patients with comorbidities such as renal dysfunction. Various modalities including second-generation non-sedating H1 antihistamines with or without corticosteroids are available for first-line treatment of ICM or non-ICM associated AU. Corticosteroids are not highly recommended due to their controversial benefit, whereas antihistamines are necessary (29). In our center, both ebastine 10 mg daily and levocetirizine 10 mg daily could control the symptoms of ICM-induced AU within several days, and ebastine showed superior overall efficacy. In contrast to this, ebastine 10 mg daily was found to be inferior to levocetirizine 5 mg for symptomatic relief of non-ICM related AU (30). The discrepancy may be reasonable as many variables must be considered, such as study types, gender ratio, ethnic difference, subjects with comorbidities, and concomitant medications other than antihistamines. Although efficacy comparisons among other antihistamines in ICM-related AU are lacking, the above data collectively imply the preference of ebastine or levocetirizine monotherapy for AU-affected patients with prophylactic methylprednisolone.

There are many limitations in the present study. The evidence on prophylaxis and treatment of AU from retrospective analysis are usually less stringent compared with prospective and randomized controlled trials. The incidence of AU may be underreported as some self-limited AU might be unnoticed. Furthermore, we could not rule out the effect of other drugs consumed, especially heparin, on the incidence of AU during CAG, but related drug hypersensitivity could be negligible judging from the rate of HSRs during computed tomography procedures without application of these drugs (1, 10, 12). A scientifically proven explanation for the difference between limited and diffuse AU could not be provided. On the whole, the current study only involved Asian patients, and hence, the evidence we provided may not be generalized to non-Asian population.

## Conculusion

Collectedly, the first large-scale systematic analysis in China demonstrated that ICM-induced AU is not uncommon in non-emergent CAG inpatients with prophylactic methylprednisolone. Preexisting allergies, renal dysfunction, and mild inflammation are high-risk factors, and antihistamine monotherapy is a favorable candidate for ICM-related AU. Future prospective investigations are needed to develop a better personalized prophylaxis and management strategy to tackle ICM-associated AU or other HSRs in high-risk patients, especially the patients who are undergoing ICM administration through the intra-arterial route.

## Data Availability Statement

The raw data supporting the conclusions of this article will be made available by the authors, without undue reservation.

## Ethics Statement

The study was approved by the Ethics Committee of the People's Hospital of China Medical University. No patient consent was needed due to a retrospective study and analysis on the medical records of the recruited patients.

## Author Contributions

YW and FH designed the research study. FY performed the analysis. BC and YW collected data and wrote the manuscript. WC made revisions to the manuscript. All authors approved the final version of the manuscript.

## Conflict of Interest

The authors declare that the research was conducted in the absence of any commercial or financial relationships that could be construed as a potential conflict of interest.
